# The Role of Autophagy and Pyroptosis in Liver Disorders

**DOI:** 10.3390/ijms23116208

**Published:** 2022-06-01

**Authors:** Huijie Zhao, Huiyang Liu, Yihan Yang, Honggang Wang

**Affiliations:** 1 Institute of Chronic Disease Risks Assessment, Henan University, Kaifeng 475004, China; zhj5696@163.com; 2 School of Basic Medical Sciences, Henan University, Kaifeng 475004, China; m15736875597@163.com (H.L.); h1323240458@163.com (Y.Y.)

**Keywords:** autophagy, pyroptosis, non-alcoholic fatty liver disease, hepatocellular carcinoma, hepatotoxicity

## Abstract

Pyroptosis is a programmed cell death caused by inflammasomes, which can detect cell cytosolic contamination or disturbance. In pyroptosis, caspase-1 or caspase-11/4/5 is activated, cleaving gasdermin D to separate its N-terminal pore-forming domain (PFD). The oligomerization of PFD forms macropores in the membrane, resulting in swelling and membrane rupture. According to the different mechanisms, pyroptosis can be divided into three types: canonical pathway-mediated pyroptosis, non-canonical pathway-mediated pyroptosis, and caspase-3-induced pyroptosis. Pyroptosis has been reported to play an important role in many tissues and organs, including the liver. Autophagy is a highly conserved process of the eukaryotic cell cycle. It plays an important role in cell survival and maintenance by degrading organelles, proteins and macromolecules in the cytoplasm. Therefore, the dysfunction of this process is involved in a variety of pathological processes. In recent years, autophagy and pyroptosis and their interactions have been proven to play an important role in various physiological and pathological processes, and have gradually attracted more and more attention to become a research hotspot. Therefore, this review summarized the role of autophagy and pyroptosis in liver disorders, and analyzed the related mechanism to provide a basis for future research.

## 1. Introduction

In the 1990s, a caspase-1-dependent and bacteria-induced cell death appeared in macrophages infected with *Salmonella typhimurium*, named pyroptosis in 2000 [1,2,3]. Pyroptosis is a kind of gasdermin(GSDM)-mediated programmed cell death, characterized by the formation of holes in the cell membrane, cytolysis, and the release of the pro-inflammatory cytokines. Pyroptosis is an important innate immune mechanism and contributes to the inflammation through releasing interleukin 1β(IL-1β), IL-18 and other inflammatory substances [4,5]. More and more evidence indicates that pyroptosis contributes to many diseases, including liver diseases [6,7]. However, the relevant mechanisms have not been fully clarified. Autophagy is an important, closely coordinated and conserved cellular pathway. This process separates proteins and damaged or aged organelles into double-membrane vesicles named autophagosomes and finally fuses with lysosomes, resulting in the degradation of isolated components [8]. Autophagy plays an important role in maintaining the balance of cell component synthesis, decomposition and reuse, and participates in various physiological processes [9]. It has been reported that autophagy is involved in many diseases, such as cancer, neurodegenerative diseases and infection/immune diseases [10,11,12,13]. Evidence shows autophagy inhibition upregulates galangin-induced pyroptosis in human glioblastoma multiforme cells [14] and promotes pneumococcus-induced pyroptosis [15], indicating that autophagy and pyroptosis are closely related and play a vital role in a variety of physiological and pathological processes. Furthermore, in recent years, autophagy and pyroptosis and their interactions have been reported to play an important role in a variety of physiological and pathological processes, and have attracted increasing attention to become a research hotspot. However, the relevant mechanism is not completely clear [16]. Therefore, in this review, we reviewed the recent progress regarding the role and the mechanism of autophagy, pyroptosis and the relationship between them in liver disorders to provide theoretical reference for future related research.

## 2. Overview of Pyroptosis

### 2.1. Characteristics and Mechanism of Pyroptosis

In 1992, when A. Zychlinsky treated macrophages with *Shigella flexneri*, pyroptosis was first found [2]. However, this was regarded as a kind of apoptosis, and it was named pyroptosis until 2000 [17,18]. Pyroptosis is a programmed cell death, significantly different from apoptosis and autophagy in cell morphology and function. It is characterized by membrane perforation mediated by gasdermin(GSDM) protein family, inflammatory factors (including IL-1β and IL-18) release and cytolysis [19]. GSDM proteins include GSDMA, GSDMB, GSDMC, GSDMD, GSDME and DFNB59. Except for DFNB59, the other GSDM family proteins all have similar N-terminal parts, which is related to the formation of the pyrolytic pores in the cell membrane. It has been reported that GSDMB, GSDMC, GSDMD and GSDME are related to pyroptosis; whether other GSDM family proteins contribute to pyroptosis remains to be studied [20,21]. Many pathological factors play an important role in pyroptosis, including cholesterol, oxidative stress and inflammatory cytokines [4]. Cholesterol is one of the important structural components of the mammalian cell membrane. It can destroy the stability of the lysosomal membrane structure and lead to lysosomal damage and cause the outflow of lysosomal contents, resulting in NLRP3 inflammasome activation and pyroptosis [22]. Reactive oxygen species(ROS) produced by oxidative stress can activate NLRP3 inflammasome, then activate caspase-1 to initiate pyroptosis [23]. Pyroptosis is a highly inflammatory cell death pattern induced by inflammatory microsomes, which depends on the activation of caspase-1 [24]. Caspase-1 cleaves IL-1β precursors into active IL-1β which recruits and activates other immune cells, promotes the synthesis of chemokines (such as IL-18), inflammatory factors (such as IL-6) and adhesion factors, and finally leads to a “cascade effect”, thus amplifying the inflammatory response [25,26].

### 2.2. Classification of Pyroptosis

**Figure 1:**
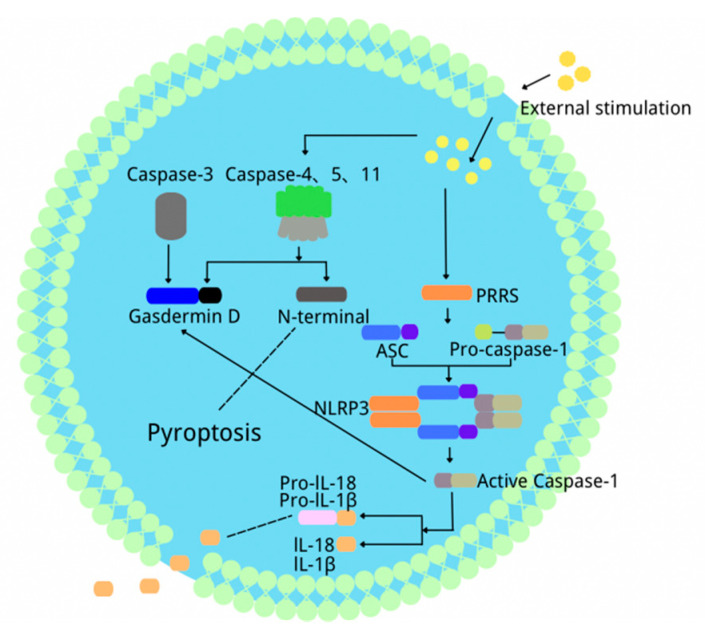
The schematic diagram of the process of three types of pyroptosis.

So far, there are three pathways leading to pyroptosis. One is the canonical pathway [27], the second is the non-canonical pathway [28], and the third is the recently discovered caspase-3-induced pyroptosis. After cells receive different stimuli, pyroptosis is initiated by different pathways but finally completed by GSDM protein [29]. Canonical pyroptosis is mediated by caspase-1 activated by the NLRP3 inflammasome. The active caspase-1 can be automatically cleaved into its CARD domain and P20/P10 dimers at the specific location. After that, the two P20/P10 dimers oligomerize to form a tetramer to cleave the specific site of GSDMD and accurately bind to the GSDMD-C domain [30]. GSDMD is cut into N-terminal fragments that can attach to the cell membrane and oligomerize to form the pyroptotic pore. Moreover, P20/P10 tetramers can cleave pro-IL-1β and pro-IL-18 induced by NF-κB signalling into their active forms. Furthermore, due to the water inflow caused by osmotic pressure, the pyrolysis pore can lead the cell to swell, and IL-1β and IL-18 can escape through GSDMD pores, thus inducing inflammation [28]. Unlike the canonical pathway, non-canonical pyroptosis depends on the activation of caspase-4/5/11 [31]. Human caspase 4/5 and mouse caspase 11 can bind to bacterial LPS and induce inflammation of cell necrosis. Like caspase-1, activated caspase-11 can also cleave GSDMD and release IL-1β and IL-18, then induce the formation of cell membrane pores. In addition, the activated caspase-11 can also promote K^+^ outflow, activate NLRP3/ASC/caspase-1 and induce cellular inflammatory response [30,32,33]. Recently, it has been found that there is also a caspase-3-dependent pyroptosis pathway. Unlike caspase-1/11/4/5, caspase-3 induces cell pore formation through cleaving GSDME and promoting the re-entry of the GSDME-N domain into the cell membrane, resulting in pyroptosis (Figure 1) [4]. The distribution and expression level of GSDME determine the cell death pattern through caspase-3 activation. When cells overexpress GSDME, activated caspase-3 will induce pyroptosis. For cells expressing a low level of GSDME, the active caspase-3 will induce apoptosis [29,34,35]. In recent years, pyroptosis has been reported to play a vital role in many diseases, including liver diseases [36,37]. However, the related mechanism is not completely clear.

## 3. Overview of Autophagy

**Figure 2:**
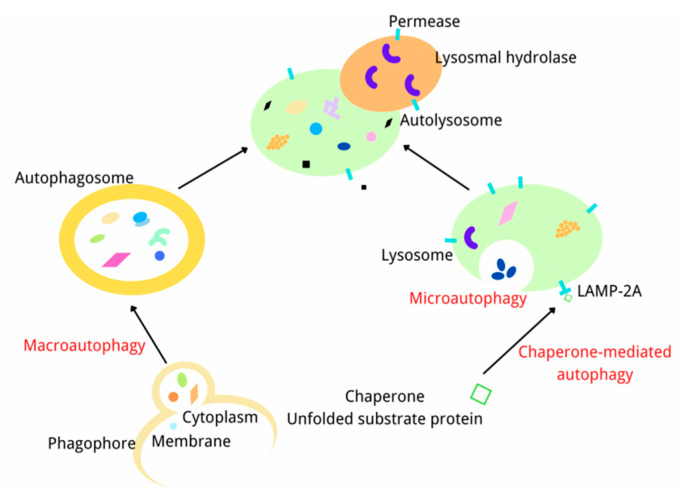
The Diagram of the process of three kinds of autophagy.

Autophagy is a self-degradation and self-sustaining process in eukaryotic cells, which plays a significant role in clearing damaged organelles, proteins or cell fragments from cells [38]. In this process, the abnormal proteins, organelles, and pathogens are wrapped in bilayers to form autophagosomes that are transferred to lysosomes for degradation [39]. At present, there are three kinds of autophagy based on the transmission pathway of proteins and organelles to lysosomes: macroautophagy, microautophagy, and chaperone-mediated autophagy [40]. Macroautophagy is responsible for the degradation of microorganisms and organelles and is the most studied autophagy. In this process, the substance to be degraded is wrapped by a double membrane vesicle to form an autophagosome then fused with the lysosome for degradation. Microautophagy does not form autophagosomes and mainly degrades cell components by invaginating and/or dividing the cytoplasm on the lysosomal membrane. Chaperone-mediated autophagy is a selective autophagy in which intracellular proteins are transported to the lysosomal chamber after binding with chaperones and then digested by lysosomal enzymes(Figure 2) [41]. As we all know, autophagy is caused by various environmental stresses, such as nutrient deficiency, hypoxia and growth factor deficiency, to eliminate the stress-induced damage and help cells to return to normal after stress relief [42]. Autophagy under physiological conditions is usually maintained at a basic level. Stress stimulation can significantly enhance autophagy, thereby eliminating abnormal proteins in cells to promote cell survival [43]. However, if autophagy is at a high level for a long time, cell death will be induced. Thus, the role of autophagy is a “double-edged sword” [44,45]. Increasing evidence indicates that dysfunctional autophagy is involved in many diseases, including liver diseases [46,47], although the relevant mechanism has not been fully studied.

## 4. The Role of Autophagy and Pyroptosis in Liver Disorders

### 4.1. The Role of Autophagy and Pyroptosis in Nonalcoholic Fatty Liver Disease

Non-alcoholic fatty liver (NAFLD) includes fatty liver, non-alcoholic steatohepatitis (NASH) and liver cirrhosis, excluding excessive drinking and viral infection. It is a clinicopathological syndrome characterized by liver fat accumulation. Because of its high incidence rate (about 20–30%) and the lack of effective clinical treatment, NAFLD has become a serious chronic disease globally [48,49,50]. NAFLD is associated with many factors, including type 2 diabetes mellitus (T2DM), insulin resistance, dyslipidaemia and hypertension, although the exact mechanism is not fully understood [51,52]. Ghrelin is a polypeptide containing 28 amino acids. It is synthesized from gastric mucosa and secreted into the blood. There are two main forms: acylated ghrelin and deacylated ghrelin [53,54]. The increasing evidence indicates that ghrelin plays an important role in NAFLD [55,56]. Silvia Ezquerro et al. showed that the circulating acylation/deacetylation ghrelin ratio and TNF-α in obese patients with NAFLD were upregulated, while the level of deacetylated ghrelin decreased. Six months after bariatric surgery, the liver function was significantly improved, and the circulating acylated/deacylated ghrelin ratio decreased. In obese patients with type 2 diabetes, ghrelin, and its acylase ghrelin O-acyltransferase(GOAT) increased, and pyroptosis, apoptosis, and compromised autophagy of liver cells increased [57]. It has been reported that TNF-α-induced hepatocyte cell death contributes to NAFLD development [58]. Thus, reducing TNF-α-induced hepatocyte cell death may become a new strategy to improve NAFLD. In HepG2 hepatocytes, the acylated and deacylated ghrelin decreased TNF-α- induced cleavage of caspase-3 and caspase-8, TUNEL positive cells, caspase-1 activation, and high-mobility group box 1(HMGB1) expression, indicating that ghrelin inhibited apoptosis and pyroptosis induced by TNF-α. In addition, the acylated ghrelin inhibited the basal and TNF-α-induced hepatocyte autophagy, which can be demonstrated by the downregulated LC3II/I ratio and upregulated p62 accumulation via AMPK/mTOR. Collectively, ghrelin reduces TNF-α-induced human hepatocyte apoptosis, autophagy, and HMGB1-mediated pyroptosis to play a protective role against hepatocyte cell death, thus preventing NAFLD progression to NASH [57]. In the above study, the impaired autophagy mediated the protective role of ghrelin in the human hepatocyte, which contradicted the previous report that ghrelin upregulated autophagy in rat hepatocytes to improve liver injury [59,60]. The reason may be related to the different stages of liver injury and remains to be clarified. It has been reported that TNF-α increases cytoplasmic HMGB1 expression to induce pyroptosis during liver failure [61]. Therefore, it can be deduced that ghrelin suppressed HMGB1-mediated pyroptosis by reducing TNF-α. In the ghrelin improvement of NAFLD, whether autophagy can regulate pyroptosis remains to be studied.

Taurine (Tau) is a sulfur-containing compound β-amino acids, which exists in many human and animal tissues, and it plays an important role in the prevention of NASH [62]. Tianming Qiu and colleagues found that arsenic trioxide(As_2_O_3_) could cause NASH, upregulated autophagy, activate NLRP3 inflammasome, increase lipid accumulation, and lead to the dysregulation of lipid-related genes. Tau dampened the inflammation, pyroptosis and autophagy induced by As_2_O_3_. In HepG2 cells, NLRP3 inflammasome activation, which was cathepsin B(CTSB)-dependent, mediated As_2_O_3_-induced pyroptosis. Moreover, the inhibition of autophagy by inhibitor suppressed As_2_O_3_-induced upregulated expression of cytosolic CTSB and subsequent LDH release, NLRP3 inflammasome activation and pyroptosis, suggesting that the increase of intracellular autophagy was related to the increase of cytoplasmic CTSB, the activation of NLRP3 inflammasome and its mediated pyroptosis induced by As_2_O_3_. In addition, Tau inhibited As_2_O_3_-induced autophagy, CTSB expression, NLRP3 inflammasome activation, and pyroptosis, and reduced LDH release. From the above, it could be deduced that Tau attenuated As_2_O_3_-induced pyroptosis through inhibiting CTSB-dependent NLRP3 inflammasome activation induced by As_2_O_3_ via suppressing As_2_O_3_-induced autophagy. Furthermore, the inhibition of NLRP3 inflammasome, autophagy, and CTSB, and Tau treatment did not reduce lipid accumulation induced by As_2_O_3_, indicating that Tau dampened As_2_O_3_-induced liver inflammation and pyroptosis by inhibiting the autophagic-CTSB-NLRP3 inflammasome pathway rather than reducing lipid accumulation [63]. Autophagic death often contributes to many liver disorders [64,65]. Similarly, in the above study, As_2_O_3_-induced autophagy leads to hepatocyte pyroptosis, which is involved in the development of NASH. In the above role of Tau in improving NASH, autophagy promotes pyroptosis through NLRP3 inflammasome. Many previous pieces of evidence have shown that autophagy is closely related to lipid metabolism [66,67,68]. Pyroptosis is also involved in lipid metabolism [69,70]; therefore, whether autophagy/pyroptosis is related to As_2_O_3_-induced liver lipid accumulation needs further study. Similar to Tau, liraglutide can also improve NASH. Liraglutide is an analogue of glucagon-like peptide-1 (GLP-1) and can improve NASH [71,72]. Xinyang Yu and colleagues used palmitic acid and lipopolysaccharide to stimulate HepG2 cells to establish a NASH model to assess the role of liraglutide, NLRP3 inflammasome and mitophagy in NASH. Liraglutide decreased lipid accumulation, suppressed NLRP3 inflammasome and pyroptosis activation, improved mitochondrial dysfunction, reduced reactive oxygen species (ROS) production, and enhanced hepatocyte mitophagy. The inhibition of mitophagy by 3-methyladenine (3-MA)/PINK1-directed siRNA dampened liraglutide suppression of NLRP3 inflammasome and pyroptosis activation, suggesting that liraglutide improved NASH by suppressing NLRP3 inflammasome and pyroptosis activation through promoting mitophagy [73]. In the above study, it can be deduced that enhancing mitophagy can alleviate NLRP3 inflammasome-mediated infammatory injury and pyroptosis of NASH. In liraglutide improvement of NASH, mitophagy inhibits pyroptosis by inhibiting NLRP3 inflammasome activation. Mitophagy can inhibit the activation of NLRP3 inflammasome by scavenging damaged mitochondria and reducing the ROS production, and then inhibit the activation of the canonical pathway of pyroptosis. Evidence indicates that LPS induces pyroptosis through a non-canonical process by activating caspase-11 [69,74]. Then, in the above study, whether mitophagy inhibits pyroptosis through caspase-11 remains to be clarified. Another study also confirmed that autophagy/pyroptosis is involved in NASH. Blueberries have been reported to improve NASH [75]; however, it is unclear which active ingredient in blueberries plays this role. Juanjuan Zhu et al. found that tectorigenin (TEC, one active ingredient in blueberries) could distinctly suppress lipid droplet formation, inflammatory mediators release, and promote cell proliferation in steatosis hepatocytes. Similarly, TEC also inhibited lipid damage and lipid accumulation induced by high-fat diets in vivo. In the NASH model of mice and cell, TEC promoted autophagy and suppressed pyroptosis and the release of inflammatory mediators. Moreover, 3-MA abolished TEC-mediated inhibition of the lipid deposition, NLRP3, and GSDEM (a marker of pyroptosis), indicating that TEC suppressed pyroptosis, NLRP3 inflammasome, and lipid deposition through promoting autophagy in the NASH model of cells. In addition, the expression of tRF-47 (a kind of tsRNAs) was upregulated by TEC. tRF-47 knockdown dampened TEC improvement of NASH in vitro through the inhibition of autophagy, activation of pyroptosis and promotion of infammatory factors release. Similarly, tRF-47 inhibition worsened the lipid deposition of NASH in vivo. Collectively, TEC ameliorated NASH by inhibiting pyroptosis through promoting autophagy via upregulating tRF-47 [76]. In the above study, enhancing autophagy suppressed pyroptosis by inhibiting NLRP3 inflammasome/GSDME in TEC improvement of NASH, which needed to be further confirmed.

**Figure 3:**
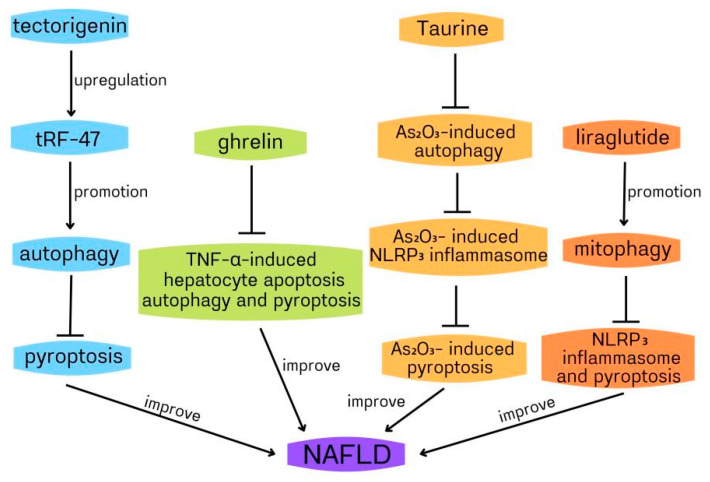
The protective role of autophagy and pyroptosis against non-alcoholic fatty liver disease (NAFLD).

In conclusion, autophagy and pyroptosis play a protective role against NAFLD (Figure 3), which will provide a new strategy for the treatment of NAFLD.

### 4.2. The Role of Autophagy and Pyroptosis in Hepatocellular Carcinoma

Hepatocellular carcinoma (HCC) is one of the most frequent primary liver cancers and the third leading cause of cancer death [77,78]. The evidence indicates that 17β-estradiol (E2) plays a protective role against HCC by activating the NLRP3 inflammasome [79]; however, the mechanism is unclear. The results of Qing Wei and colleagues showed that E2 induced NLRP3 inflammasome activation, evidenced by increased expression levels of caspase 1 and IL-1β in HCC cells. E2 also reduced the viability and increased the mortality rate of HepG2 cells. At the same time, caspase 1-specific inhibitor YVAD-cmk significantly reversed the E2 cytotoxic effect, indicating that E2 induced HCC cell death through activating the NLRP3 inflammasome. Further experiments showed that E2 notably downregulated autophagy in HCC cells, which was reversed by YVAD-cmk, indicating that E2 inhibition of autophagy was mediated by NLRP3 inflammasome. Additionally, the inhibition of autophagy by 3-MA significantly promoted E2-induced pyroptosis, which was reversed by YVAD-cmk, suggesting that autophagy negatively regulated caspase-1-dependent pyroptosis. Summarily, E2 induced NLRP3 inflammasome-caspase 1-dependent pyroptosis through inhibiting autophagy. Autophagy negatively regulates pyroptosis through NLRP3 inflammasome [80]. In the above study, autophagy inhibition promotes the pyroptosis of HCC cells, thus providing a new idea for HCC treatment by regulating autophagy.

### 4.3. The Role of Autophagy and Pyroptosis in Hepatotoxicity

Patulin is a mycotoxin produced by many common fungi in fruit and vegetable products. It has been reported that patulin induces hepatotoxicity [81,82,83]. Qian Chu et al. found that patulin promoted pyroptosis and NLRP3 inflammasome-mediated inflammation, evidenced by the upregulated expression levels of NLRP3, IL-1β, IL-18, pro-caspase-1, cleaved caspase-1 GSDMD, and cleaved GSDMD in mouse livers. Similarly, in HepG2 cells, patulin also induced pyroptosis and NLRP3 inflammasome activation, while treatment with NLRP3 inhibitor MCC950 or cathepsin B inhibitor downregulated the levels of NLRP3, caspase-1 and IL-1β, indicating that NLRP3 inflammasome/cathepsin B mediated patulin-induced pyroptosis. Caspase-1 inhibitor Ac-YVAD-cmk reduced the levels of GSDMD and IL-1β in HepG2 cells, which confirmed that patulin-induced pyroptosis was dependent on NLRP3 inflammasome. Furthermore, autophagy inhibitor 3-MA dampened patulin-induced induction of cytoplasmic cathepsin B expression, NLRP3 inflammasome activation, pyroptosis and inflammation. Collectively, patulin promoted pyroptosis perhaps through upregulating autophagy/NLRP3 inflammasome/cathepsin B in the liver. The above study showed that patulin could induce autophagy, reduce the stability of the lysosomal membrane, activate cathepsin B and then activate NLRP3 inflammasome, thus finally causing pyroptosis. That is to say, autophagy positively regulates pyroptosis through the NLRP3 inflammasome [84].

Benzo[a]pyrene (BaP) is a common polycyclic aromatic compound which is easy to be produced in the processing of petroleum and fatty food. It is a strong carcinogen and has strong immunotoxicity and reproductive toxicity [85,86,87]. As the main metabolic organ of BaP intake, the ability of the liver to metabolize BAP is much stronger than that of other organs, so the hepatotoxicity caused by BAP is also stronger than that of other organs [88,89,90]. Li Yuan and colleagues showed that BaP promoted HL-7702 cell death, upregulated the intracellular levels of ROS and inhibited HL-7702 cell growth by blocking the cell cycle in the S phase. BaP induced pyroptosis is evidenced by the increase of LDH and NO release, and the electrical conductivity of HL-7702 cells. Meanwhile, BaP also upregulated the protein expression levels of procaspase-1, caspase-1, IL-1β and IL-18. Since caspase-1 and inflammatory factors are important markers of pyroptosis activation, it could be deduced that BaP induced cell death by promoting pyroptosis. Moreover, in HL-7702 cell, BaP enhanced autophagy, and the inhibition of autophagy by 3-MA notably suppressed the release of NO and LDH, the upregulation of electrical conductivity, and the expression levels of pyroptotic marker proteins (caspase-1, IL-1β, IL-18), indicating that BaP induced pyroptosis by promoting autophagy. Futhermore, the pyroptosis inhibitor Ac-YVAD-CM also significantly abolished BaP-promoted autophagic cell death, evidenced by the increase of autophagic vacuoles and the upregulated expression of LC3-II and Beclin-1. Summarily, BaP induced HL-7702 cell death by promoting autophagy and pyroptosis simultaneously. In addition, Autophagy and pyroptosis promote each other in HL-7702 cells [91]. ROS has been involved in autophagy and pyroptosis [92]. In the above study, BaP increased ROS level and induced autophagic cell death and pyroptosis in HL-7702 cells, ROS may mediate the positive relationship between autophagy and pyroptosis.

## 5. Conclusions

In this review, we summarized the role of autophagy and pyroptosis in liver disorders as follows: (1) ghrelin plays a protective role against NAFLD by decreasing TNF-α-induced human hepatocyte autophagy and HMGB1-mediated pyroptosis; (2) Tau inhibits As_2_O_3_-induced pyroptosis through inhibiting CTSB-dependent NLRP3 inflammasome activation via suppressing autophagy in NASH; (3) liraglutide ameliorates NASH through inhibition of NLRP3 inflammasome and pyroptosis activation via promoting mitophagy; (4) tectorigenin improves NASH through inhibition of pyroptosis by promoting autophagy via upregulating tRF-47; (5) 17β-estradiol promotes NLRP3 inflammasome-caspase 1-dependent pyroptosis by inhibiting autophagy; (6) patulin induces pyroptosis perhaps through autophagy/NLRP3 inflammasome/cathepsin B in the liver; (7) BaP induces HL-7702 cell death by promoting autophagy and pyroptosis simultaneously (Table 1). These results indicate that autophagic death and pyroptosis can lead to hepatocyte death, play an important role in liver disorders, and may be important targets for treating various liver diseases.

It has been reported that autophagy negatively regulates pyroptosis, and the mechanisms can be summarized as follows. One is that autophagy inhibits pyroptosis by eliminating damage-associated molecular patterns (DAMPs) and pathogen-associated molecular patterns (PAMPs). Another is that autophagy inhibits pyroptosis by inhibiting the basic components in pyroptosis [16]. In this review, autophagy regulates caspase-1-mediated canonical pyroptosis through NLRP3 inflammasome and ROS in the liver, which belongs to the second mechanism mentioned above. Whether autophagy can regulate pyroptosis through other mechanisms needs to be further studied.

It can be seen from this review that in the liver, autophagy negatively regulates pyroptosis in some cases, and autophagy and pyroptosis exist in a mutually exclusive manner. In other cases, they promote each other. This may be due to different types of cell stimulation or stimulation time because different stimuli or stimuli exposure time may lead to different autophagy and pyroptosis. The above reasons need to be further studied. At present, most existing studies on the role of autophagy and pyroptosis in the liver are in vitro. Still, there is a lack of corresponding in vivo research, which makes the basis of the research results vulnerable. Therefore, many in vivo experiments are required in the future to further verify the existing results. In addition, the role of autophagy and pyroptosis in the liver and the relationship between them have not been clearly studied and need to be further clarified in the future. Moreover, autophagy, apoptosis, NLRP3 inflammasome, and pyroptosis are closely related, so the role and relationship of the above four in liver diseases is a topic worthy of study in the future.

It is believed that with the in-depth development of relevant research, autophagy/pyroptosis will provide a new strategy for the treatment of various liver diseases.

## Figures and Tables

**Table 1:** The summary of the role of autophagy and pyroptosis in liver disorders.

The Type of Liver Disorder	The Role of Autophagy and Pyroptosis	Experimental Model	Reference
non-alcoholic fatty liver disease (NAFLD)	ghrelin plays a protective role against NAFLD by decreasing TNF-α-induced human hepatocyte autophagy and HMGB1-mediated pyroptosis	liver biopsies of NAFLD patients and human hepG2 hepatocytes	[57]
non-alcoholic steatohepatitis(NASH)	Tau inhibits As_2_O_3_-induced pyroptosis by inhibiting CTSB-dependent NLRP3 inflammasome activation via suppressing autophagy	NASH model of mice/human hepG2 hepatocytes	[63]
NASH	liraglutide ameliorates NASH through inhibition of NLRP3 inflammasome and pyroptosis activation via promoting mitophagy	NASH model of mice/human hepG2 cells	[73]
NASH	tectorigenin improves NASH through inhibition of pyroptosis by promoting autophagy via upregulating tRF-47	NASH model of mice/human hepG2 cells	[76]
hepatocellular carcinoma(HCC)	17β-estradiol promotes NLRP3 inflammasome-caspase 1-dependent pyroptosis by inhibiting autophagyd	HCC cells	[80]
hepatotoxicity	patulin induces pyroptosis perhaps through autophagy/NLRP3 inflammasome/cathepsin B in liver	mice/human HepG2 cells and L02 cells	[84]
liver injury	BaP induces HL-7702 cell death by promoting autophagy and pyroptosis simultaneously	HL-7702 cells	[91]

## Data Availability

Not applicable.
